# Knowledge of safe sex and sexually transmitted infections among high school students, Vientiane Prefecture, Lao PDR

**DOI:** 10.1080/16549716.2020.1785159

**Published:** 2020-08-03

**Authors:** Khonesavanh Inthavong, Le Thi Hai Ha, Le Thi Kim Anh, Vanphanom Sychareun

**Affiliations:** a Faculty of Public Health, University of Health Sciences, Vientiane, Lao PDR; b Hanoi University of Public Health, Vietnam; c Faculty of Social Sciences, Behavior and Health Education, Ministry of Education and Training, Ministry of Health, Hanoi University of Public Health, Vietnam; d Faculty of Fundamental Sciences, Department of Biostatistics, Ministry of Health, Hanoi University of Public Health, Vietnam

**Keywords:** LEARN: Sexual Reproductive Health, ANC and Nutrition, Adolescence, knowledge, family planning, sexual education, sexually transmitted infections

## Abstract

**Background:**

Adolescent knowledge of safe sex and sexually transmitted infections (STIs) can reduce the risk of STIs as well as unplanned pregnancies.

**Objective:**

To describe the knowledge of safe sex and STIs and to identify related factors among high school students in Vientiane Prefecture, Lao PDR.

**Method:**

This was an analytical cross-sectional study conducted at one high school from January to February 2019. A self-administered questionnaire was used to collect information from respondents. The questionnaires were completed by 337 respondents who were selected by stratified random sampling. The data collected were entered into and analysed using EpiData and Stata 13.0 software. Descriptive and inferential statistics were applied to determine the factors associated with knowledge of safe sex and STIs.

**Results:**

The results showed that nearly half of the participants (49.5%) had a good knowledge of safe sex and 51.9% of the respondents had a good knowledge of STIs. Significant positive associations were shown between knowledge of safe sex by students living with other people, those who had studied family planning and had religious beliefs reflecting acceptance to using birth control. Other factors positively associated with knowledge of STIs were students being in Grade 10, and who had studied STIs including HIV/AIDS.

**Conclusion:**

In this study, approximately half of the participants were aware of safe sex and had knowledge of STIs. There is a need to have comprehensive sexual education, particularly emphasising family planning, STIs, and HIV/AIDS for all grades in school.

## Background

Knowledge of safe sex and STIs is vitally important for adolescents who need to be imbued with a comprehensive awareness of how to avoid unsafe sex, STIs, and teenage pregnancies [1]. During the adolescent period, teenagers are at a high risk from a number of negative health consequences associated with early and unsafe sexual activities, including STIs and unintended pregnancies [2].

Previous studies have demonstrated that many adolescents are involved in sexual activities that elevate their risk of having reproductive morbidity, including unwanted pregnancies, abortions, and STIs because of a lack of basic knowledge about reproductive biology and preventive methods [3]. Regarding the knowledge of STIs, 93% of the adolescents in a study from Vietnam did not know any symptoms of STIs, 50% could not identify any cause of STIs and 76% did not know that STIs could be prevented [4]. Adolescents have been reported by WHO to not know how to avoid a pregnancy or access contraceptives, including emergency contraception [5].

Globally, each year estimated 333 million new cases of curable STIs occur, with the highest rates among 20–24 year olds, followed by 15–19 year olds [6]. Besides that, more than one million STIs are acquired every day. Each year, it is estimated there are 357 million new STIs [7]. Moreover, estimated 23 million girls aged 15–19 years have unintended pregnancies in developing regions [8].

Lao PDR is a lower-middle-income country situated in southeast Asia. The country has a high adolescent fertility rate with 10.9% of Lao adolescents giving birth by the age of 15–18 and 4.7% of the adolescents having a live birth before the age of 15 [9]. A study in Vientiane reported 33.4% of the participants aged 15–19 years had engaged in pre-marital sexual intercourse and 62.7% of the adolescents had their first sexual experience before the age of 15. In the six months prior to the survey, 48.5% of the adolescents reported not using condoms during sexual intercourse [10]. In addition, 2.9% of the male and 0.5% of the female adolescents had multiple sexual partners [9]. This is of concern as previous research from the period 2010–2012 showed the incidence of HIV among 15 to 24-year-olds equated to 16.7% (2010), 18.8% (2011), and 15.9% (2012) of all new cases in each respective year [11]. It was also reported that while legally restricted, 23.2% of 15–24 year old women had an abortion [12].

Based on the Sustainable Development Goals (SDGs), adolescents’ sexual and reproductive health is a priority on the global agenda within the UN aim to enable low- and middle-income countries to reach the SDGs [13]. Thus, the global health agenda reiterates the need for adolescents to have higher levels of knowledge about sexual health, be more able to make informed decisions about their sexual health and more likely to have protected sex, while avoiding STIs and unplanned pregnancies [14].

In recent years, several researchers have concentrated on knowledge of safe sex and STIs, but without focusing on factors that affected levels of knowledge of safe sex and STIs among adolescents [15]. There are few studies that have gathered data from in-school adolescents in Lao PDR on sexual knowledge and its determinants. The aim of this study was to describe the knowledge of safe sex and STIs and associated factors among high school students in Vientiane City. Understanding adolescents’ knowledge of safe sex and STIs can help design appropriate educational programmes that support adolescents’ sexual decision-making [7, 15–17] and findings from such research are important in planning preventive and treatment strategies.

## Methods

### Study design and setting

This study had an analytical cross-sectional design and used a structured questionnaire. The study was conducted at one high school in Vientiane city from January to February 2019. At the time of the study, the number of students studying in grades 10 to 12 was about 1,507 and of those, 896 were female. The age range was 14 to 20 years.

### Sample size and sampling

The target population of the study was students studying in grades 10–12. The formula of estimating a proportion with specified relative precision formula was applied in order to sample size determination. The expected proportion was the prevalence of having good knowledge of safe sex and STIs among high school students. Due to lack of evidence about this proportion from previous studies, 50% was used as an estimate, with a 5% significance level, a margin of error of 5% and an expected non-response rate of 10%. This gave an overall sample size of 337. Of the total 1,507 potential participants in the school, 21% were in grade 10, 38% in grade 11, and 41% in grade 12. The sample was stratified by these proportions to target 71 participants in grade 10, 128 in grade 11 and 138 in grade 12.

### Measurements

A self-administrated structured questionnaire was used to collect information about knowledge of safe sex and STIs, socio-demographic characteristics and other variables such as characteristics of school, family, peers, and religious factors.

The independent variable included socio-demographic factors such as age, gender, ethnicity, grade, and family structure. The questions about communication with family and peers sought answers using categories ‘Never’, ‘Rarely’, ‘Sometimes’, and ‘Often’ during their lifetime [16,17]. The question related to schooling gave information about students attending school, sexual education topics, time allocated to study and providing adequate sexual education in school [17,18]. The questionnaire for the sources of knowledge for safe sex and STIs was divided into two parts covering safe sex and STIs, and each part contained seven questions using multiple choice answers to categorise answers with yes (marked as 1) or no (marked as 0). These questions touched topics such as where students got information about family planning and where they got information about STIs from the mass media [18]. The question about the participant’s religious factors included religious belief, the importance of religion, attendance at religious services and their religion’s level of acceptance for sexual intercourse before marriage and the use of birth control [18].

The dependent variable to knowledge of safe sex was measured by 11 positive questions and 2 negative questions [17–20] and the tools for the questionnaire about a knowledge of STIs consisted of 33 items regarding an understanding of the types, causes, routes, symptoms of STIs and types of prevention for STIs [17,18,21,22], with three answer choices (Yes, No, Don’t know or Unsure) for each question. They were given one point for correct answers and zero points for ‘Don’t Know’ or ‘Unsure’ and incorrect answers. This gave a total score for knowledge of safe sex and STI questions with a normal distribution, so the mean was used as an indicator to categorise levels of knowledge, good knowledge > mean and poor knowledge **≤** mean [1,23,24].

The outcome variables of this study for both levels of knowledge for safe sex and STIs were classified into two categories (low knowledge and good knowledge) and the mean was used as an indicator to categorise knowledge levels. Hence, good knowledge ≥ mean and poor knowledge < mean [1,23,24].

### Data collection

The questions were initially prepared in English and then translated into Lao, after which they were piloted with 30 respondents in a non-study school before data collection. The value of Kuder–Richardson 20 (KR-20) was utilised to test the reliability of achievement test with dichotomous choices. The coefficient of KR20 for the knowledge of safe sex and STIs was 0.77 and 0.93, respectively. During data collection, the investigators explained to the respondents the research objectives and methods and obtained informed consent before they completed the self-administered questionnaire. All questionnaires were checked for completeness and consistency during the fieldwork.

### Data analysis

This study used EpiData to enter the data and Stata 13.0 for analysis. Descriptive statistics were used for frequencies and percentages of the independent and outcome variables. Tests of significance using univariate and multivariate logistic regression were performed to calculate odds ratios which were used to test associations between independent and outcome variables, with 95% confidence intervals for estimating the precision of the odds ratios, with 95% confidence intervals of adjusted odds ratios excluding unity reflecting significant associations.

### Ethical approval and consent to participate

Ethical clearance was obtained from the University of Health Sciences in Vientiane, Lao PDR (Approval Number: 103/18, Date 12/12/2018) and the Hanoi University of Public Health (Approval Number: 018-464/DD.YTCC Date 12/12/2018). Participation was voluntary and informed consent was obtained from the participants and parental consent was also obtained. Participants had the right to withdraw at any time and the information collected from the respondents was kept strictly confidential. To protect the confidentiality of participants, the names of respondents were not included in questionnaires, and information collected from respondents was stored confidentially. The research objectives, method conditions, and potential risks were explained to all respondents before the questionnaires were completed.

## Results

In total, 337 students from grades 10 to 12 participated in this study. Participants were aged 14–20 years and the mean age was 16 (SD = 1.09), 66.5% of the participants were female. Most of the participants were ethnic Lao (83.9%) and 76.8% of the participants lived with their parents. Three-quarters of the participants came from families with no more than five people (Table 1). The main source of knowledge about family planning cited was Facebook (84.5%), followed by films or television (78.9%). The two most common sources of information for STIs including HIV/AIDs were also from films or television (85.1%) and Facebook (84.8%).

**Table 1.** Socio-demographic and other characteristics of participants.

Variables	Number (n = 337)	Percentage (%)
**Age (Years)** Mean and SD = 16.47 ± 1.088, Median = 16, Min = 14, Max = 20		
14–16 years old	172	51.0
17–20 years old	162	48.9
**Sex**		
Female	224	66.4
Male	113	33.5
**Ethnicity**		
Lao	283	83.9
Hmong	20	5.9
Khamu	2	0.5
Tai	29	8.6
Other	3	0.8
**Grade of study**		
Grade 10	71	21.0
Grade 11	128	27.9
Grade 12	138	40.9
**Living with**		
Parents	259	76.8
Single mother	27	8.0
Single father	8	2.3
Single mother and step father	4	1.1
Single father and step mother	2	0.5
Brother/Sister	4	1.1
Cousin	18	5.3
Housemate in dormitory/rented house	15	4.4
**People in family. Mean and SD = 4.95 ± 1.55, Median = 5, Min = 2, Max = 13**		
2–5	254	75.3
>5	83	24.6

### Knowledge of participants about safe sex and STIs

Participant knowledge about safe sex is shown in Table 2. The majority of participants knew correct and consistent condom use resulted in safer sex. Less than a quarter of participants answered correctly however about falling pregnant during the regular menstrual cycle.

**Table 2.** Knowledge of students about safe sex.

No.	*Variables*	Male (n = 113)		Female (n = 224)		Total (n = 337)	
		N	%	N	%	N	%
**Knowledge of students about safe sex**							
1	It is better to have only one partner for a sexual relationship	72	63.7	156	69.6	228	67.6
2	A condom should be used correctly and consistently for a safe sex purpose	98	86.7	186	83.0	284	84.2
3	A condom cannot prevent STIs/HIV infection	65	57.5	95	42.4	160	47.4
4	Have had sexual intercourse with only one partner without HIV	48	42.4	103	45.9	151	44.8
5	Prevention of AIDS is no sex with risky persons	76	67.2	161	71.8	237	70.3
6	Sex during the menstrual cycle cannot protect you from pregnancy	29	25.6	52	23.2	81	24.0
7	Even first time sexual intercourse can cause pregnancy	88	77.8	169	75.4	257	76.2
8	Safe sex (i.e. sex which is free from the risk of unwanted pregnancy and STD/AIDS)	48	42.4	99	44.2	147	43.6
9	Condoms can help prevent pregnancy	95	84.0	137	61.1	232	68.8
10	Birth control pill can help prevent pregnancy	61	53.9	123	54.9	184	54.6
11	Birth control injection can help prevent pregnancy	45	39.8	119	53.1	164	48.6
12	Abstinence can help prevent pregnancy	79	69.9	167	74.5	246	73.0
13	Intrauterine Device (IUD) can help prevent pregnancy	50	44.2	104	46.4	154	45.7

***Presented only yes answer***

Most participants correctly identified HIV/AIDS as an STI and just over a third knew that viruses caused STIs. The majority knew that sexual intercourse and shared needle use were routes for the transmission of STIs. Just over aone-third of participants knew a discharge from the penis or vulva was a sign or symptom of infection. A few participants regarded weakness as a sign or symptom of an STI. Regarding the prevention of STIs, most participants knew it was good to get tested before marriage or starting a new relationship. Finally, a large majority was aware that the consistent use of condoms was a safe way of preventing STIs (Table 3).

**Table 3.** Knowledge of students about STIs.

No.	*Variables*	Male (n = 113)		Female (n = 224)		Total (n = 337)	
		N	%	N	%	N	%
**Knowledge of students about STIs**							
	Type of STIs						
1	Syphilis	45	39.8	101	45.0	146	43.3
2	Influenza	84	74.3	178	79.4	262	77.7
3	Gonorrhea	78	69.0	164	73.2	242	71.8
4	Chlamydia	51	45.1	103	45.9	154	45.7
5	HPV	36	31.8	62	27.6	98	29.0
6	Meningitis	64	56.2	139	62.0	203	60.2
7	HIV/AIDS	103	91.1	215	95.9	318	94.3
8	Herpes	25	22.1	40	17.8	65	19.2
	Cause of STIs						
9	Bacteria	42	37.1	76	33.9	118	35.0
10	Virus	56	49.5	80	35.7	136	40.3
11	Fungus	15	13.2	17	7.5	32	9.5
12	Bad hygiene of woman	17	15.0	63	28.1	80	23.7
13	Bad hygiene of man	19	16.8	49	21.8	68	20.1
14	Using unclean water	58	51.3	140	62.5	198	58.7
	Route of STIs						
15	Sexual intercourse	97	85.8	199	88.8	296	87.8
16	Blood transfusion	75	66.3	154	68.7	229	67.9
17	Sharing needle	86	76.1	185	82.5	271	80.4
18	Sharing clothes, belongings	88	77.8	188	83.9	276	81.9
19	Sharing foods	85	75.2	184	82.1	269	79.8
20	Mother to child	72	63.7	172	76.7	244	72.4
	Sign and symptom of STIs						
21	Abdominal pain	19	16.8	27	12.0	46	13.6
22	Discharge from penis/vulva	47	41.5	80	35.7	127	37.6
23	Itching in genital area	40	35.4	75	33.4	115	34.1
24	Burning pain on urination	30	26.5	42	18.7	72	21.3
25	Pain during intercourse	26	23.0	44	19.6	70	20.7
26	Loss of weight	41	36.2	84	37.5	125	37.0
27	Weakness	63	55.7	120	53.5	183	54.3
	The way of preventing STIs						
28	Consistent condom use	93	82.3	180	80.3	273	81.0
29	Monogamy	80	70.8	179	79.9	259	76.8
30	Get tested before marriage/before starting new relationships	102	90.2	209	93.3	311	92.2
31	Condoms	94	83.1	154	68.7	284	73.5
32	Birth control pills offer excellent protection	47	41.5	87	38.8	134	39.7
33	Once you have had STIs and have been cured, you can’t get it again	50	44.2	113	50.4	163	48.3

***Presented only yes answer***

Regarding the 13 questions on knowledge of safe sex, a score was generated with a mean of 7.49, SD ± 2.4. Knowledge of safe sex was categorised into two groups on each side of the mean (Table 4).

Referring to the 33 questions about knowledge of STIs, a score was generated with a mean of 17.30, SD ± 5.1. Knowledge of STIs was categorised into two groups on each side of the mean (Table 4).

### Multivariable analysis

The multivariable logistic regression model is presented in Table 5. Variables with a p-value of <0.005 in the univariate analyses were entered into the multivariate analysis, namely age, sex, grade, living arrangement, following a religion accepting the use of birth control, studies of family planning methods and STIs. The significant factors associated with knowledge of safe sex were students living with people other than their parents (AOR = 2.5, 95% CI 1.1 to 5.3), who had studied family planning (AOR = 1.7, 95% CI 1.0 to 2.9) and following a religion that accepted the use of birth control (AOR = 1.7, 95% CI 1.0 to 2.9). Significant factors associated with knowledge of STIs were that higher grade students were less knowledgeable (Grade 12 AOR = 0.3, 95% CI 0.1 to 0.6 and Grade 11 AOR = 0.4, 95% CI 0.2 to 0.8), while those who had studied family planning (AOR = 1.8, 95% CI 1.0 to 3.1) and STIs including HIV/AIDS (AOR = 5.1, 95% CI 1.9–13.5) were more knowledgeable.

**Table 4.** Level of knowledge on safe sex and STIs.

Level of knowledge (score)	Number (n = 337)	Percentage (%)
Knowledge of safe sex		
Poor knowledge (≤ mean)	170	50.4
Good knowledge (>mean)	167	49.5
Mean = 7.49 and SD ±2.438, Median = 7, Min = 0,Max = 13		
Knowledge of STIs		
Poor knowledge (≤ mean)	162	48.0
Good knowledge (>mean)	175	51.9
Mean = 17.30 and SD ± 5.095, Median = 18, Min = 0,Max = 29

**Table 5.** Results in multivariate logistic regression analysis for the factors associated with students’ knowledge of safe sex and STIs.

Variables	Knowledge of safe sex and STIs (n = 337)						
	Crude Adjusted						
	COR	95%CI		AOR	95%CI		P-value
		Lower	Upper		Lower	Upper	
**Knowledge of safe sex**							
**Live with**							
With parent(s)				1			
With other(s)	2.6	1.2	5.5	2.5	1.1	5.3	0.017*
**Study topic about family planning**							
No	1			1			
Yes	1.8	1.1	2.9	1.7	1.0	2.9	0.035*
**Religion accepts use of birth control**							
No	1			1			
Yes	1.8	1.1	2.9	1.7	1.0	2.9	0.025*
**Knowledge of STIs**							
**Grade**							
Grade 10	1			1			
Grade 11	0.5	0.3	1.0	0.4	0.2	0.8	0.009*
Grade 12	0.3	0.2	0.7	0.3	0.1	0.6	0.002*
Study t**opic about family planning**							
No	1						
Yes	2.2	1.3	3.6	1.8	1.0	3.1	0.033*
**Study topic about STIs including HIV/AIDS**							
No	1			1			
Yes	5.8	2.3	14.6	5.1	1.9	13.5	0.001*

*Statistically significant odds ratios (P < 0.05)

## Discussion

Adolescents with higher levels of knowledge about sexual health are more likely to have protected sex, reducing the risk of STIs and unplanned pregnancies [1,2,25–27]. Most students in this study had good knowledge about safe sex, particularly that the correct and consistent use of condoms can reduce the risk of most STIs including HIV and unintended pregnancies. A study in Lao PDR found that adolescents considered condoms to be generally the safest method of preventing unplanned pregnancy and STIs [28]. Condoms have been widely promoted in education and social marketing campaigns in Lao PDR and are likely to contribute to students’ knowledge of condoms. Positively, almost all participants knew to get an STI/HIV test before starting a new sexual relationship. Nevertheless, similar to other studies in the region, in Vietnam [23] and Indonesia [1], while students had some knowledge about STIs, misconceptions about causation and symptoms were evident.

While respondents in grades 11 and 12 reported significantly less knowledge about STIs than those in grade 10, this may reflect improvements over time in education and information programmes about STIs. Knowledge as to when pregnancy is most likely to occur in a woman’s menstrual cycle was also generally poor.

The study showed factors associated with higher knowledge of safe sex were living with people other than parents, studying family planning and belonging to a religion that accepted the use of birth control. While parents may be an important source of information on sexual and reproductive health at some stages of adolescence, living more independently may reflect other opportunities for gaining knowledge and experience in these matters [29–41].

Studying family planning was generally associated with improved use of contraceptives. School is often seen as an important source for building sexual health knowledge [16,42–44]. Some research has found curriculum-based sex education to be moderately associated with a decrease in adolescents’ risky sexual behaviours (e.g. unprotected sex) [16,42]. Despite sexual education and family planning being included in the school curriculum in higher secondary school, in the present study, knowledge about family planning and STIs, including HIV was mainly derived from Facebook and films or television. The reason for this is not known but it may relate to the teaching methods, teacher competency or trust in sexual information provided by teachers [45].

Religion can be an important factor affecting knowledge about safe sex [18,46–48]. In this study, being of a religion that accepted using of birth control was associated with knowledge of safe sex.

### Limitations

One of the limitations is the sample was drawn from one school and cannot therefore be considered as representative of all the schools in Lao PDR. Additionally, due to the nature of the cross-sectional study design, causes and effects could not be explored extensively. Furthermore, the study was based on self-reporting and could subject to social desirability bias. In addition, it is possible there might have been some issues regarding the tone of language in the questionnaire which may have caused confusion for some respondents and they had no immediate support to clarify certain questions.

## Conclusion

Overall, adolescents included in this study had a generally low knowledge of safe sex and STIs. There is a need for comprehensive sexual education particularly in relation to content knowledge about family planning and STIs and HIV/AIDS. There is also a need for future research studies in other schools, particularly those in rural areas which, in the context of a rapidly developing country such as Lao PDR, may provide a stark contrast to urban areas. In addition, qualitative research combined with quantitative research may provide a more nuanced perspective.

## Paper context

Adolescents are considered a key population for understanding knowledge related to unsafe sex. This study indicated that while adolescents have a general awareness of safe sex and sexually transmitted infections, several misconceptions were evident. Improving access to knowledge of safe sex and prevention of STIs is important in adolescence for preventing the negative consequences associated with early childbearing and STIs, including HIV/AIDS, among adolescents.
